# Urgent intraoperative endovascular stent placement to resolve acute hepatic or portal venous obstruction during liver surgery: a case series

**DOI:** 10.1186/s40792-020-01093-4

**Published:** 2021-01-06

**Authors:** Yutaro Kato, Atsushi Sugioka, Masayuki Kojima, Junichi Yoshikawa, Yoshinao Tanahashi, Sanae Nakajima, Akira Yasuda, Gozo Kiguchi, Yuichiro Uchida, Toshihiro Yasui, Tatsuya Suzuki, Hokuto Akamatsu, Ryota Hanaoka, Hiroyuki Nagata, Ryoichi Kato, Ichiro Uyama

**Affiliations:** 1 Department of Surgery, Fujita Health University, 1-98, Dengakugakubo, Kutsukake-cho, Toyoake, Aichi 470-1192 Japan; 2 Department of Pediatric Surgery, Fujita Health University, Toyoake, Japan; 3 Department of Radiology, Fujita Health University, Toyoake, Japan

**Keywords:** Endovascular stenting, Vascular reconstruction, Self-expandable metallic stent, SEMS, Interventional radiology, Hepatic vein, Portal vein, Liver resection, Liver transplantation

## Abstract

**Background:**

Acute obstruction of the hepatic vein (HV) or the portal vein (PV), particularly when it occurs during liver surgery, is potentially fatal unless repaired swiftly. As surgical interventions for this problem are technically demanding and potentially unsuccessful, other treatment options are needed.

**Case presentation:**

We report two cases of acute, surgically uncorrectable HV or PV obstruction during liver resection or living donor liver transplantation (LDLT), which was successfully treated with urgent intraoperative placement of endovascular stents using interventional radiology (IVR). In Case 1, a patient with colonic liver metastases underwent a non-anatomic partial hepatectomy of the segments 4 and 8 with middle hepatic vein (MHV) resection. Additionally, the patient underwent an extended right posterior sectionectomy with right hepatic vein (RHV) resection for tumors involving RHV. Reconstruction of the MHV was needed to avoid HV congestion of the anterior section of the liver. The MHV was firstly reconstructed by an end-to-end anastomosis between the MHV and RHV resected stumps. However, the reconstruction failed to retain the HV outflow and the anterior section became congested. Serial trials of surgical revisions including re-anastomosis, vein graft interposition and vein graft patch-plasty on the anastomotic wall failed to recover the HV outflow. In Case 2, a pediatric patient with biliary atresia underwent an LDLT and developed an intractable PV obstruction during surgery. Re-anastomosis with vein graft interposition failed to restore the PV flow and elongated warm ischemic time became critical. In both cases, the misalignment in HV or PV reconstruction was likely to have caused flow obstruction, and various types of surgical interventions failed to recover the venous flow. In both cases, an urgent IVR-directed placement of self-expandable metallic stents (SEMS) restored the HV or PV perfusion quickly and effectively, and saved the patients from developing critical conditions. Furthermore, in Cases 1 and 2, the SEMS placed were patent for a sufficient period of time (32 and 44 months, respectively).

**Conclusions:**

The IVR-directed, urgent, intraoperative endovascular stenting is a safe and efficient treatment tool that serves to resolve the potentially fatal acute HV or PV obstruction that occurs in the middle of liver surgery.

## Background

Concomitant vascular resection with reconstruction is often required during liver resection for hepatobiliary tumors involving major vessels [1]. Failure of vascular reconstruction may lead to life-threatening liver insufficiency and unstable systemic circulatory status. In liver transplantation, unsatisfactory graft perfusion due to failed reconstruction of the hepatic vein (HV) or the portal vein (PV) may lead to graft and ultimately, patient loss. In living donor liver transplantation (LDLT), the small sizes of the diameters of recipient and graft PVs and the positioning of the partial liver graft, may affect the patency and alignment of anastomoses. The stricture or twisting at the HV–caval or interpositional vein graft anastomosis must also be prevented during LDLT [2].

Such intraoperative acute HV or PV obstruction requires urgent surgical repair including direct re-anastomosis, implementation of vascular grafts and removal of intravascular thrombi. However, as such surgical attempts may result in failure, other options need to be devised as this complication is life-threatening. In this report, we present two cases of successful intraoperative endovascular placement of self-expandable metallic stents (SEMSs) by interventional radiology (IVR) for critical HV or PV obstruction that occurred in the middle of liver surgery.

## Case presentation

### Case 1

**Fig. 1:**
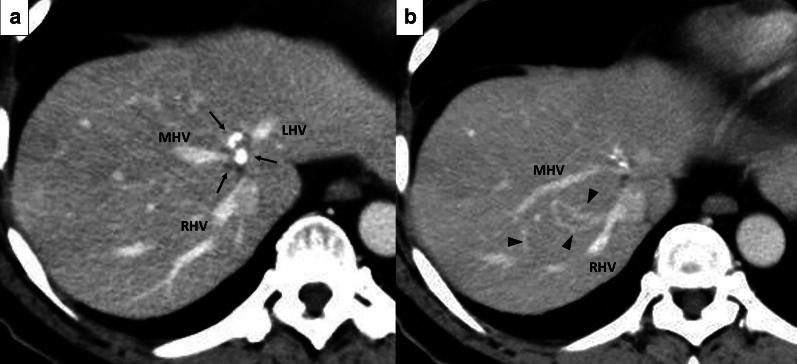
Preoperative computed tomography images (Case 1). **a** A metastatic tumor (arrows) involved the root of the MHV and left hepatic vein (LHV). The root of the RHV was intact. **b** Veno-venous communications between the MHV and RHV (arrowheads) that had developed as a result of MHV obstruction by tumor invasion

The patient was a 58-year-old male with synchronous, multiple colonic liver metastases which had remarkably responded to chemotherapy. He had a tumor at the segments 4 to 8 (S4/8) of the liver, involving the root of the middle hepatic vein (MHV) (Fig. 1a). The outflow from the right anterior section of the liver (RAS) was drained into the right hepatic vein (RHV) via the peripheral veno-venous communications that had developed between the MHV and RHV (Fig. 1b). We planned a non-anatomic resection of S4/8 with a combined circumferential resection of MHV for this tumor. Moreover, we needed to perform an extended right posterior sectionectomy (ERPS) with RHV resection to remove other tumors involving the RHV. Reconstruction of the MHV was mandatory to avoid hepatic venous congestion of the remnant RAS, as its draining vein, the RHV, would be removed during the ERPS.

**Fig. 2:**
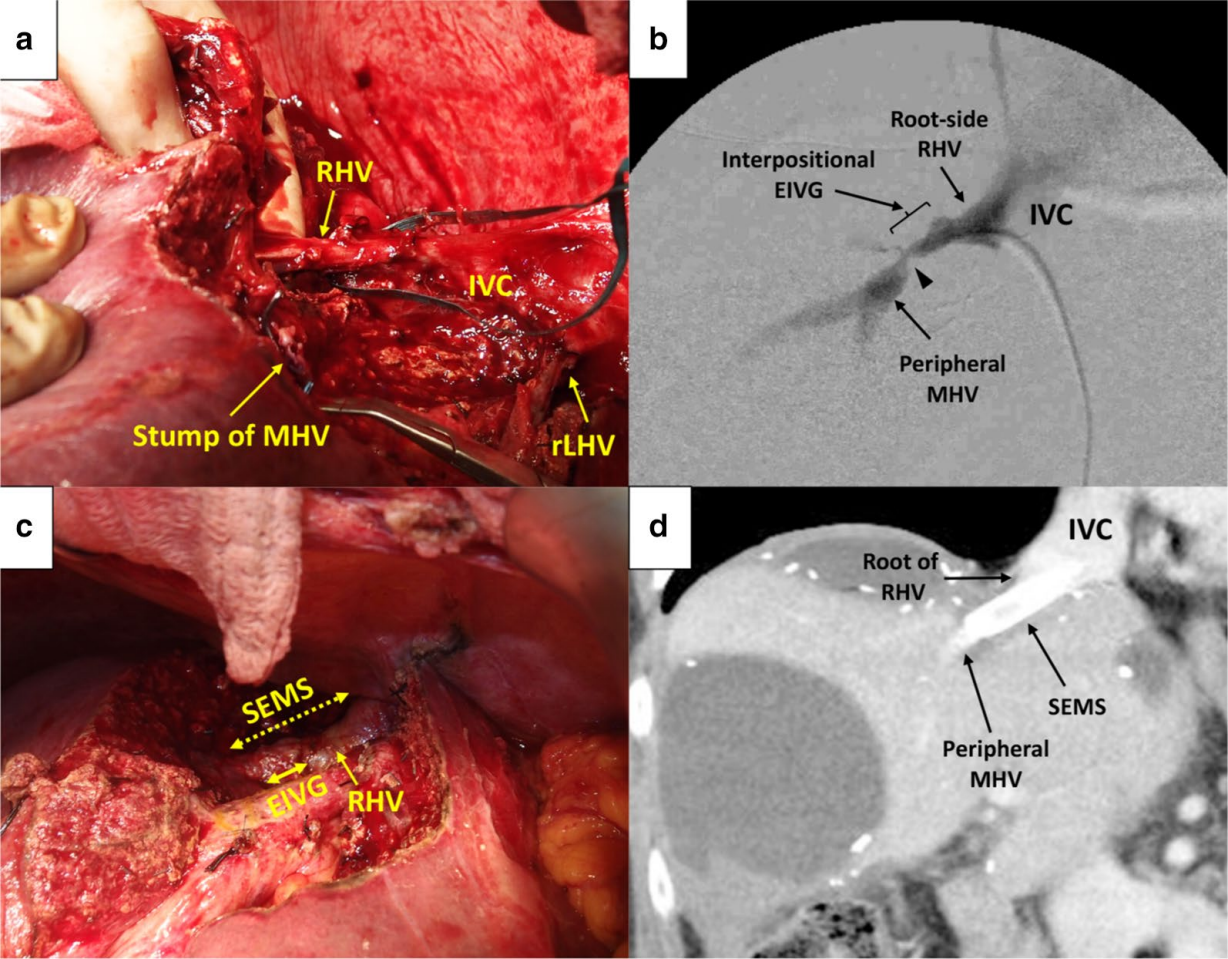
Intraoperative findings (**a**–**c**) and a postoperative computed tomography (CT) image (**d**) (Case 1). **a** A picture showing the operative field after a segment S4/8 non-anatomic hepatectomy with combined resection of the root-side part of the MHV. Note the clamped stump of the MHV. Inferior vena cava (IVC) and the RHV were exposed. The LHV was already partially resected and reconstructed (rLHV) after an anatomic segment 3 resection for another tumor. **b** Intraoperative hepatic venogram showing a severe stricture at the anastomosis between the interpositional EIVG and peripheral MHV (arrowhead). **c** A picture showing the operative field after deployment of SEMS (dotted two-way arrow) inside the HV reconstruction from the RHV to the peripheral MHV across the anastomosis using an EIVG (solid two-way arrow). **d** A CT image at 8 months postoperatively showing a fully patent SEMS with sufficient MHV outflow

Here, we focused on the reconstruction of the MHV after its resection during an S4/8 partial hepatectomy and the subsequent EPRS. The distal resected stump the MHV was about 60 mm far away from the divided stump of the MHV root (Fig. 2a). A 30-mm-long proximal section of the RHV was preserved at its root. The diameters of the MHV and RHV stumps were 6 mm and 8 mm, respectively. After a right-sided mobilization of the liver to improve the reconstructive alignment, we performed the MHV reconstruction under inflow occlusion, by an end-to-end anastomosis with continuous sutures between the MHV and RHV stumps.

Immediately after restarting the hepatic inflow, the remnant RAS became swollen with persistent oozing from the cut liver surface, suggesting hepatic venous congestion. Doppler ultrasonography (US) confirmed no flow inside the reconstructed MHV and a regurgitating flow in the right anterior branch and trunk of the PV. We instantly revised the anastomoses, firstly by a direct end-to-end re-anastomosis, secondly with an interposition of autogenous right external iliac vein graft (EIVG) between the stumps of the RHV and MHV, and finally by a vein graft patch repair on the EIVG–MHV anastomosis. However, all these revisions failed to restore the HV outflow. As further surgical attempts appeared to be impossible due to the fragility and small diameter of the peripheral stump of the MHV, we thought of repairing the failed reconstruction by IVR in the operating room.

An urgent hepatic venogram by direct HV catheterization from the left femoral vein showed that the EIVG–MHV anastomosis had a firm stricture due to twisting, which was seen to have caused the outflow obstruction (Fig. 2b). We placed an 8 mm in diameter, 60 mm in length SEMS inside the reconstructed MHV covering the area from the RHV root to the intrahepatic part of the MHV across the EIVG–MHV anastomosis (Fig. 2c). This procedure was successful in resuming the HV outflow, and the regurgitating PV flow disappeared on Doppler US.

The postoperative course was complicated by temporary liver failure, but the patient gradually recovered and was discharged from the hospital on postoperative day 81. The placed stent had been patent for 32 months after stenting until its obstruction by recurrent tumor invasion (Fig. 2d).

### Case 2

**Fig.3:**
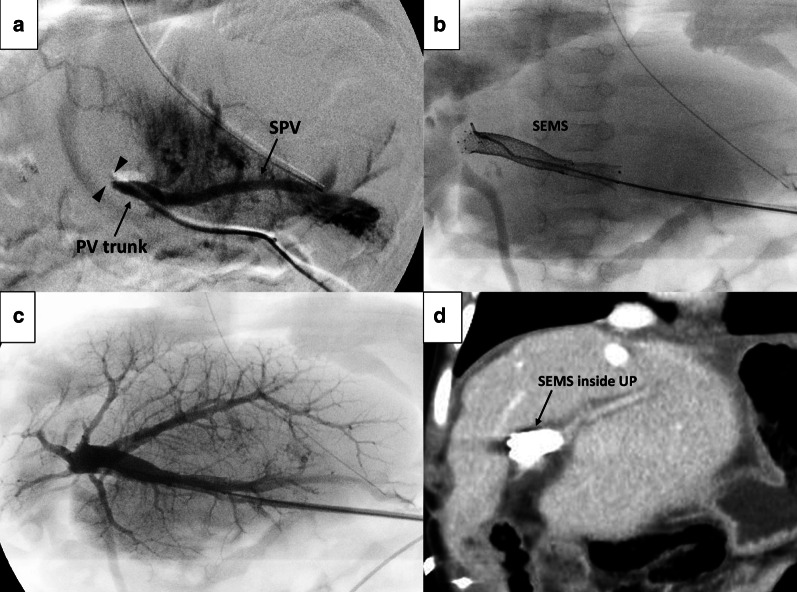
Intraoperative portal venograms (**a**–**c**) and a postoperative computed tomography (CT) image (**d**) (Case 2). **a** A portal venogram showing a complete obstruction of the PV anastomosis (arrowheads) and a regurgitating flow to the splenic vein (SPV). **b** Two overlapping SEMSs were implemented to dilate the portal vein anastomosis. **c** The portal venogram after the successful stenting. The PV flow was completely restored from the PV trunk to the intrahepatic portal vein branches. **d** A CT image at 3 postoperative months showing patent SEMS and sufficient portal venous flow in the liver graft

The patient was a 6-month-old boy with biliary atresia who underwent LDLT using the left lateral section of his mother as a graft. The PV of the donor liver was anastomosed to the recipient PV trunk in an end-to-end fashion using the branch-patch technique due to the small diameter of the recipient PV trunk. As a sufficient PV flow was not obtained, the anastomosis was immediately revised with an interposition of the donor’s grafted ovarian vein, but the PV flow still did not recover. An emergency intraoperative portal venogram via the middle colic vein showed an almost complete obstruction at the anastomotic site (Fig. 3a). As further surgical revisions were unlikely to restore the PV flow, we placed two SEMSs inside the reconstructed PV, overlapped each other to cover the area from the porto-splenic junction to the umbilical portion (UP) of the grafted PV (Fig. 3b). After successful endovascular stenting, sufficient intra-PV flow was obtained (Fig. 3c). The patient had no problems with PV flow during the postoperative course (Fig. 3d) and was discharged in good health on posttransplant day 69. The stent was still fully patent at 44 months from the SEMS placement.

## Discussion

Acute obstruction of the (reconstructed) major HV or the PV during liver surgery, whatever the cause, is an urgent status condition that may lead to fatal hemodynamic complications, unless immediate correction is achieved. In such a situation, surgical revision may not always be successful, because the secondary procedures are generally more difficult than the first reconstruction, due to the inferior condition of the veins, altered position of the organs, exacerbated bleeding tendency and unstable systemic circulatory state. Both patients in this report suffered difficult situations in which a variety of surgical revisions resulted in failure. Under these tough circumstances, an IVR-directed, urgent endovascular placement of SEMS completely resolved these potentially fatal complications which occurred in the operating room. Furthermore, the treatment was quick, safe and highly effective, with a sufficient duration of patency, although the patients may need life-long anticoagulants to prevent in-stent thrombosis formation.

The probability of such critical intraoperative vascular complications that need urgent stenting during liver surgery is unknown; there were no previous reports on this issue. In our institute, during the last 20 years between 2000 and 2019, we performed 65 hepatectomies in conjunction with HV and/or inferior vena cava (IVC) reconstruction, 114 hepatectomies with concomitant PV reconstruction, and 74 LDLTs (unpublished data). During this period, we needed an urgent intraoperative HV or PV stent placement in the middle of liver surgery in three cases (3/253, 1.2%) including two reported in this article. Although the probability of surgically uncorrectable vascular obstruction during liver surgery is low as shown above, we need to consult the IVR team preoperatively in cases at high risk for HV reconstruction failure. At the moment, we consider that the high-risk cases are those where the hepatic vein to be resected and reconstructed drains one or more liver sections and at the same time, a small-diameter (≤ 7 mm) and long-distance (≥ 4 cm) end-to-end anastomosis is planned.

Several etiologies may underlie the perfusion problems in our cases. In Case 1, in the first and second anastomoses, although the surgical techniques, the vein diameters and liver mobilization seemed to be appropriate, the reconstruction had a significant tension. So, we next interposed a vein graft to relieve the tension, but the peripheral end of the EIVG–MHV anastomosis created a twisting. This may be because the directions of the MHV and EIVG orifices did not conform well. Then, we patched a vein graft on the anterior wall of the EIVG–MHV anastomosis to enlarge the anastomotic orifice to lessen the effect of twisting. However, this revision was also unsuccessful. Collectively, the most crucial factor influencing the perfusion failure was that we inappropriately selected the RHV as the opponent vein for the anastomosis with the MHV stump, which may have resulted in the misalignment of reconstruction. Considering the alignment problem in this case, it might have been a better practice to anastomose the stump of MHV and a newly opened hole on the IVC with a long interposed vein graft in the first place. In Case 2, the size-mismatched large-size liver graft was an important detrimental factor, because the porta hepatis of the graft was significantly deviated to the right side from the appropriate position, which may have produced an inadequate alignment of the PV reconstruction.

Previous studies described the use of IVR-directed endovascular stenting for the postoperative acute or chronic stricture or obstruction of the reconstructed HV or PV [3–5]. These studies reported favorable post-stenting results with technical success rates of over 80%. Furthermore, a preoperative endovascular stenting for a tumor abutting HV or PV was shown to protect liver function and raise its resectability [6]. On the other hand, Shin et al. reported that 7 out of 10 right-lobe LDLT recipients who received endovascular stent placement for occlusion of interposed MHV grafts were treated within 24 h after transplantation [3]. Ko et al. reported 2 pediatric cases of stent placement for PV occlusion within 1–2 days after LDLT [4]. It is possible that the patients in these series should have been treated with SEMS intraoperatively rather than postoperatively, considering the short intervals between surgery and stent placement. To our knowledge, there is one previous report describing an intraoperative endovascular placement of SEMS for PV obstruction after failed reconstruction during hepatectomy [7]. However, there is no previous report on intraoperative stent placement for obstructed HV reconstruction during liver resection or for failed HV or PV reconstruction during LDLT.

Balloon angioplasty (BA) is another useful option for either PV or HV stenosis after LDLT and liver resection [8–10]. In our case series, we did not choose BA because of the potential risk of rupture of the fresh anastomoses and the expected insufficiency to improve the strictures by balloon dilatation alone. The safe minimum interval between vascular anastomosis and the following BA is unclear and thus the anastomotic rupture during balloon dilatation is a major concern, particularly when it is performed within 1–2 h after vascular anastomosis. Furthermore, a PV or an HV stricture due to a tension on the anastomosis, kinking of the anastomosed veins or inadequate alignment of the venous reconstruction is unlikely to improve solely by BA, because this method does not effectively eliminate these etiologies [4]. Therefore, under these circumstances, endovascular stent placement should be chosen rather than BA.

Upon acute obstruction of PV during surgery, creation of a mesenterico-portal bypass between a mesenteric vein and the intrahepatic portal vein system is a potential method to relieve portal hypertension and restore the PV flow into the liver. For this purpose, a permanent bypass by a jumping vascular graft anastomosis [11] or a temporary bypass using a tube [12] is potentially useful. In Case 2, creation of a Meso-Rex bypass between the superior mesenteric vein (SMV) and the liver graft UP using autogenous veins such as the internal jugular vein might have been an option [11]. However, the SMV was atrophic in our patient and we could not find other mesenteric veins suitable for creation of the bypass. In Case 2, an intraoperative placement of SEMS was selected as a safer and less invasive procedure than an urgent creation of a mesenterico-portal bypass.

## Conclusions

An IVR-directed, intraoperative emergency endovascular stenting with SEMS is a useful and recommended treatment option to resolve acute and potentially fatal obstruction of the HV or the PV during liver surgery, particularly after failure of other surgical corrections.

## Data Availability

The datasets are not available for public access due to patient privacy concerns, but are available from the corresponding author on reasonable request.

## Abbreviations

BA: Balloon angioplasty
CT: Computed tomography
ERPS: Extended right posterior sectionectomy
EIVG: External iliac vein graft
HV: Hepatic vein
IVC: Inferior vena cava
IVR: Interventional radiology
LDLT: Living donor liver transplantation
LHV: Left hepatic vein
MHV: Middle hepatic vein
PV: Portal vein
RAS: Right anterior section of the liver
RHV: Right hepatic vein
rLHV: Reconstructed left hepatic vein
SEMS: Self-expandable metallic stent
UP: Umbilical portion of the left portal vein
US: Ultrasonography
